# Olaparib Combined with DDR Inhibitors Effectively Prevents EMT and Affects miRNA Regulation in *TP53*-Mutated Epithelial Ovarian Cancer Cell Lines

**DOI:** 10.3390/ijms26020693

**Published:** 2025-01-15

**Authors:** Patrycja Gralewska, Łukasz Biegała, Arkadiusz Gajek, Izabela Szymczak-Pajor, Agnieszka Marczak, Agnieszka Śliwińska, Aneta Rogalska

**Affiliations:** 1 Department of Medical Biophysics, Institute of Biophysics, Faculty of Biology and Environmental Protection, University of Lodz, 141/143 Pomorska Street, 90-236 Lodz, Poland; patrycja.gralewska@biol.uni.lodz.pl (P.G.); lukasz.biegala@edu.uni.lodz.pl (Ł.B.); arkadiusz.gajek@biol.uni.lodz.pl (A.G.); agnieszka.marczak@biol.uni.lodz.pl (A.M.); 2 Department of Nucleic Acid Biochemistry, Medical University of Lodz, 251 Pomorska Street, 92-213 Lodz, Poland; izabela.szymczak@umed.lodz.pl (I.S.-P.); agnieszka.sliwinska@umed.lodz.pl (A.Ś.)

**Keywords:** DDR inhibitors, EMT, ovarian cancer, PARPi, miRNA

## Abstract

Epithelial ovarian cancer (EOC) remains a leading cause of gynecologic cancer mortality. Despite advances in treatment, metastatic progression and resistance to standard therapies significantly worsen patient outcomes. Epithelial–mesenchymal transition (EMT) is a critical process in metastasis, enabling cancer cells to gain invasive and migratory capabilities, often driven by changing miRNA expression involved in the regulation of pathological processes like drug resistance. Targeted therapies like PARP inhibitors (PARPi) have improved outcomes, particularly in *BRCA*-mutated and DNA repair-deficient tumors; however, resistance and limited efficacy in advanced stages remain challenges. Recent studies highlight the potential synergy of PARPi with DNA damage response (DDR) inhibitors, such as ATR and CHK1 inhibitors, which disrupt cancer cell survival pathways under stress. This study investigated the combined effects of olaparib with ATR and CHK1 inhibitors (ATRi and CHK1i) on migration, invasion, and EMT-related protein expression and miRNA expression in ovarian cancer cell lines OV-90 and SKOV-3. The results demonstrated enhanced cytotoxicity, inhibition of migration and invasion, and modulation of miRNAs linked to metastasis. These findings suggest that combination therapies targeting DNA repair and cell cycle pathways may offer a novel, more effective approach to managing advanced EOC and reducing metastatic spread.

## 1. Introduction

Ovarian cancer (OC) remains one of the most lethal gynecologic cancers, with a 5-year survival rate that has remained stagnant at around 30% for the past three decades [1]. Approximately 90% of malignant ovarian tumors originate from epithelial cells. Epithelial ovarian cancer (EOC) is further classified into several histologic subtypes, including serous cystadenocarcinoma (42%), mucinous cystadenocarcinoma (12%), endometrioid carcinoma (15%), undifferentiated carcinoma (17%), and clear-cell carcinoma (6%) [2,3]. Key prognostic factors for ovarian cancer include clinical stage, histological grade, residual disease volume, and, more recently, homologous recombination proficiency. Advanced-stage diagnoses often lead to metastatic disease—the spread of cancer cells from the original tumor to distant sites—and limited treatment options. Standard treatment typically involves surgical debulking followed by chemotherapy [3]. Although most patients initially respond well to these treatments, nearly all relapse, with metastases, which significantly worsens prognoses in OC patients and ultimately leads to fatal outcomes [4,5]. Epithelial–mesenchymal transition (EMT) is a process where cells shift from an epithelial to a mesenchymal state. This transition enables cancer cells to gain invasive and migratory abilities, making EMT a critical step for metastasis. Recent research has also highlighted a strong link between the tumor microenvironment and EMT. Conditions commonly present in the tumor microenvironment—such as hypoxia, inflammation, and oxidative stress—are powerful inducers of EMT [6]. These environmental signals activate EMT-promoting transcription factors like Snail, Slug, Twist, and Zeb1/2, which reduce the expression of epithelial markers such as E-cadherin [7].

The development of targeted therapies, including poly (ADP-ribose) polymerase (PARP) inhibitors (PARPi) such as olaparib, has advanced treatment possibilities, especially for patients with *BRCA* mutations and DNA repair deficiencies [8]. However, resistance to these therapies and cancer metastasis present major obstacles to long-term treatment efficacy [9]. Moreover, PARP inhibition in cancer cells may upregulate the homologous recombination (HR) repair pathway to maintain cell survival [10]; thus, ongoing research has uncovered promising synergies between PARPi and other DNA damage response (DDR) inhibitors, especially ataxia telangiectasia and Rad3-related (ATR) and checkpoint kinase 1 (CHK1) inhibitors [11]. ATR and CHK1 play crucial roles in maintaining cell survival under stress by managing DNA damage repair [12,13]. Inhibiting these pathways can heighten the vulnerability of cancer cells, especially when used alongside PARPi, which may then enhance treatment efficacy and potentially extend survival in patients with resistant or advanced OC.

Furthermore, recent research has focused on microRNAs (miRNAs) associated with OC that play a strong regulatory role in various cellular processes. The initial results support a possible correlation between miRNAs and cancer development, and changes in miRNA expression have been reported in various types of cancer and in chemotherapy-resistant OC [14]. In OC, some miRNAs are positively related to malignancy, including aspects such as the progression of tumors and EMT. However, the entire regulatory environment of miRNAs in OC pathogenesis has not been fully addressed. Thus, we postulated that aberrantly expressed miRNAs—whether over-expressed tumorigenic miRNAs or under-expressed protective miRNAs—contribute to the migration and invasion of OC.

By targeting molecular pathways involved in DNA repair and cell cycle regulation, PARP, ATR, and CHK1 inhibitors may also influence processes like cell migration and invasion, which are central to metastatic progression and alter different miRNA expression. Understanding the interplay between these therapies and metastatic behavior may unlock new strategies not only to shrink primary tumors but also to prevent cancer spread. This study investigated the potential of combined treatments with olaparib and DDR pathway inhibitors—ATR (ATRi, A) and CHK1 inhibitors (CHK1i, C)—to enhance cytotoxic effects, reduce metastatic behaviors, and understand the miRNA expression after treatment in OC cell lines OV-90 and SKOV-3. The findings suggest that combination treatment may lead to a new, more targeted, and effective strategy that controls and potentially limits the spread of OC and improves the clinical outcomes of patients with advanced EOC.

## 2. Results

### 2.1. Inhibitors of the ATR/CHK1 Pathway Synergistically Increased the Cytotoxicity of Olaparib in OC Cell Lines

**Figure 1:**
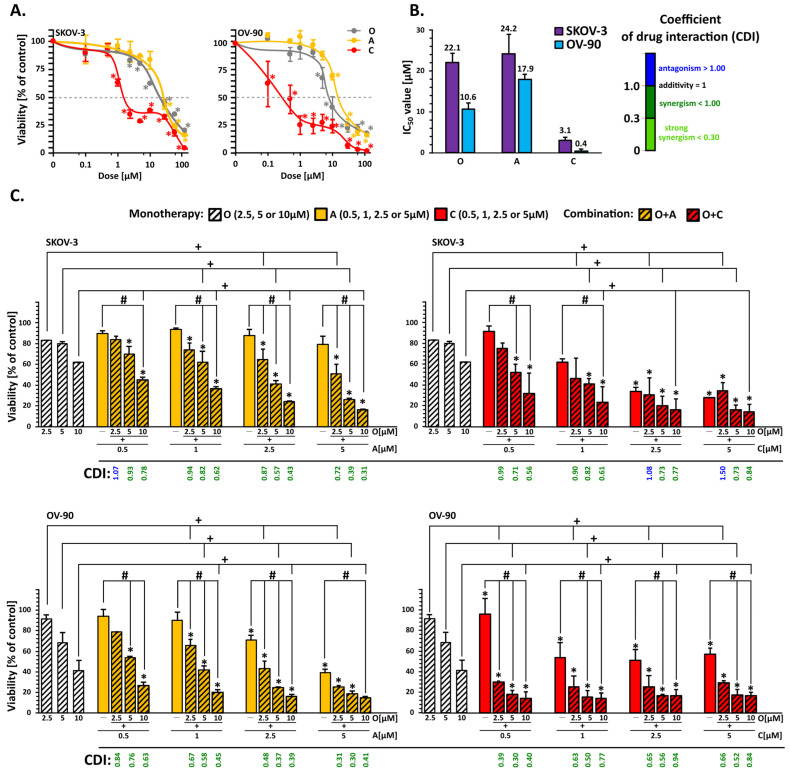
Olaparib in combination with CHK1i or ATRi decreased the viability of OC cells more effectively than PARPi alone. (**A**) Cell viability after treatment with O (PARPi), A (ATRi), or C (CHK1i) in SKOV-3 and OV-90 cell lines, at increasing concentrations for 5 days, was assessed by the MTT assay (* *p* < 0.05). (**B**) IC_50_ values of the tested inhibitors after the 5-day treatment. (**C**) The effect of olaparib combinations with A or C at different ratios, after 5 days of treatment, was evaluated by the MTT assay. * Statistically significant differences between cells incubated with the compound compared with the control cells (*p* < 0.05). + Statistically significant changes between cells incubated with O and combination treatment (O+A; O+C) (*p* < 0.05). # Statistically significant differences between the cells incubated with A or C and combination treatment (O+A; O+C) (*p* < 0.05).

To evaluate the cytotoxic effects and sensitivity of OC cell lines, a five-day treatment with increasing concentrations (up to 120 µM) of the compounds was conducted (Figure 1A). Olaparib exhibited concentration-dependent cytotoxicity, demonstrating greater efficacy in OV-90 cells than in SKOV-3, with IC_50_ values of 10.6 µM and 22.1 µM, respectively (Figure 1B). Notably, PARPi treatment did not result in complete cell death in either cell line, leaving approximately 20% of the cells viable. Similarly, CHK1i and ATRi alone were more cytotoxic to OV-90 cells.

To identify the optimal PARPi to ATRi or CHK1i ratio, multiple-concentration combination ratios were tested (Figure 1C). Based on cytotoxicity data for the individual inhibitors, three olaparib concentrations (2.5, 5, and 10 µM) and four ATRi or CHK1i concentrations (0.5, 1, 2.5, and 5 µM) were selected. To determine whether the effect of combined treatment is synergistic, the coefficient of drug interaction (CDI) was calculated. All tested combinations in OV-90 and most in SKOV-3 acted synergistically, significantly reducing cell viability (Figure 1C).

Combinations of 10 μM PARPi, 5 μM ATRi, and 2.5 μM CHK1i led to a decrease in cell viability to the level of 16.9% and 16.5% in the SKOV-3 cell line and 14.8% and 9.5% in the OV-90 cell line, respectively. These synergistic combinations were further assessed and validated in short-term treatment studies.

Additionally, the effects of these inhibitors on the mRNA expression levels of *PARP1*, *ATR*, and *CHEK1* were evaluated after 48 h of treatment at 4 µM concentrations. No significant changes in mRNA levels were observed (Figure S1B).

### 2.2. Migration of OC Cells Inhibited or Delayed by Combination Treatment with PARPi + CHK1i or PARPi + ATRi

**Figure 2:**
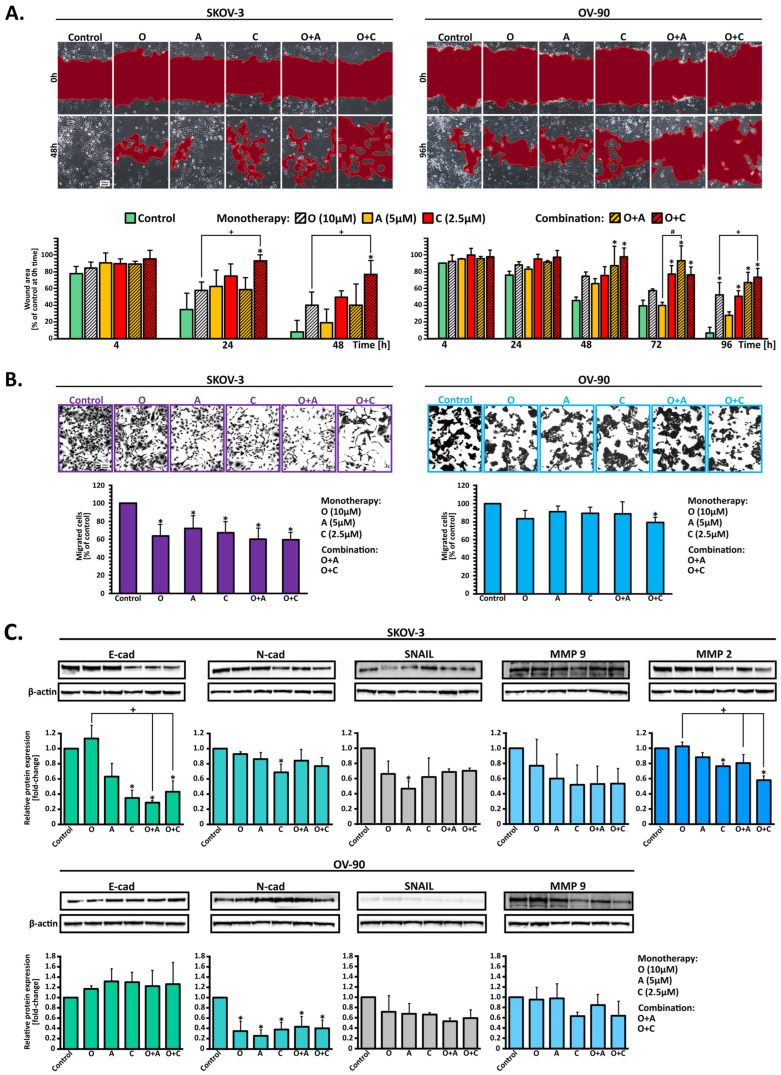
Olaparib combined with ATRi or CHK1i synergizes to affect the migration and invasion of SKOV-3 and OV-90 cells. (**A**) Representative images of the cells from the wound healing assay, acquired immediately after scratching (0 h) and 48 h (SKOV-3) or 96 h (OV-90) later, examined under an inverted phase-contrast microscope (Olympus IX70, Japan) (scale bar = 100 μm). Cellular migratory ability is presented as the percentage of wound closure. Each bar represents mean ± SD. * Statistically significant differences between cells incubated with the inhibitor’s treated cells and control cells (*p* < 0.05). + Statistically significant changes between cells incubated with O and combination treatment (O+A; O+C) (*p* < 0.05). # Statistically significant differences between the cells incubated with A or C and combination treatment (O+A; O+C) (*p* < 0.05). (**B**) Representative images of invading cells after incubation with the inhibitor compounds for 48 h. The scale bar represents 100 μm. Invasive cells were stained with crystal violet and subsequently extracted, and the absorbance values were measured. The percentage of migrated cells was estimated using the same formula as MTT. The results are expressed as mean ± SD (* *p* < 0.05). (**C**) Representative Western blot images and relative expression of MMP2, MMP9, E-cadherin, N-cadherin, and Snail in SKOV-3 and OV-90 cells. * indicates statistically significant differences between cells incubated with the compound compared with control cells (*p* < 0.05); + indicates statistically significant differences between cells incubated with O alone and the combination treatments (O+A; O+C) (*p* < 0.05).

Cancer cell migration is essential for distant metastasis, requiring cells to rearrange their cytoskeleton and propel themselves forward to invade and move through different tissues. To assess cell migration, a scratch assay was performed, measuring the ability of cells to repopulate a wounded area within a monolayer. Images were captured at various time points: 4, 24, and 48 h for SKOV-3 cells, and 4, 24, 48, 72, and 96 h for OV-90 cells. Untreated SKOV-3 cells exhibited high motility, completely repopulating the cleared area within 48 h. In contrast, migration was significantly inhibited by O+C combination treatment, leaving approximately 80% of the area uncovered (Figure 2A). Treatments with olaparib or CHK1i alone resulted in 40% and 50% uncovered areas, respectively, while O+A treatment left approximately 50% of the wound area uncovered. OV-90 cells showed similar properties, with combination treatments being more effective; PARPi and CHK1i left 74% of the wounded area uncovered after 96 h, while treatment with these inhibitors alone left about 50% uncovered (Figure 2A). These findings highlight the efficacy of combination treatments in inhibiting cell migration.

Invasion assays further demonstrated that the invasion of SKOV-3 cells was markedly reduced by both combination treatments, resulting in approximately 40% inhibition of the cell’s ability to invade (Figure 2B). In OV-90 cells, the CHK1i and PARPi combination was slightly more effective than O+A, leaving 80% and 88% of the invasive cells, respectively.

High matrix metalloproteinase (MMP) expression contributes to poor prognosis, likely due to its role in drug resistance [15] and increased metastasis or invasion [16,17]. Western blot analysis was performed to establish whether the tested inhibitors altered the MMP2 and MMP9 expression levels after 72 h of treatment (Figure 2C). Negligible MMP2 protein levels were found in OV-90 cells, regardless of treatment. In SKOV-3 cells, after treatment with the O+C combination, a 0.6-fold-change decrease in MMP2 expression was found. MMP9 expression in both cell lines demonstrated a declining tendency following CHK1i, PARPi + ATRi, and PARPi + CHK1i treatments, with SKOV-3 cells showing greater sensitivity to these treatments (Figure 2C).

To investigate potential resistance mechanisms, ABCB1/MDR1 mRNA and protein levels were examined after treatment with the tested inhibitors at 4 µM concentrations. In OV-90 cells, mRNA levels increased modestly (1.6- to 1.9-fold) after 48 h of treatment with olaparib, ATRi, and PARPi + CHK1i (Figure S1B). However, Western blot analysis revealed no significant increase in MDR1 protein expression at 24 or 48 h, suggesting that early resistance is not associated with MDR1 upregulation (Figure S1A).

Furthermore, a key EMT event is the “cadherin switch”, characterized by decreased E-cadherin and increased N-cadherin expression as cancer progresses [18]. In SKOV-3 cells, N-cadherin levels decreased significantly (0.7-fold) after CHK1i treatment, with similar reductions observed for combination treatments. Conversely, E-cadherin levels unexpectedly declined following CHK1i, O+A, and O+C treatments (Figure 2C). In OV-90 cells, an increasing trend in E-cadherin levels was found (1.2- to 1.3-fold) across all treatments, while N-cadherin expression was consistently reduced 2.5 times (0.3- to 0.4-fold change). Both cell lines exhibited reduced expression of the transcription factor Snail after all treatments, further supporting the impact of the tested inhibitors on EMT progression.

### 2.3. Olaparib Combined with ATR/CHK1 Pathway Inhibitors Alters the Expression of miRNAs in OC Cell Lines

For RT-qPCR miRNA analysis, U6 snRNA and RNU48 with an acceptable small intra- and inter-group variation in expression in both cell lines (SD of 0.11–0.48) were used as endogenous control genes for the normalization of target miRNA expression levels.

**Figure 3:**
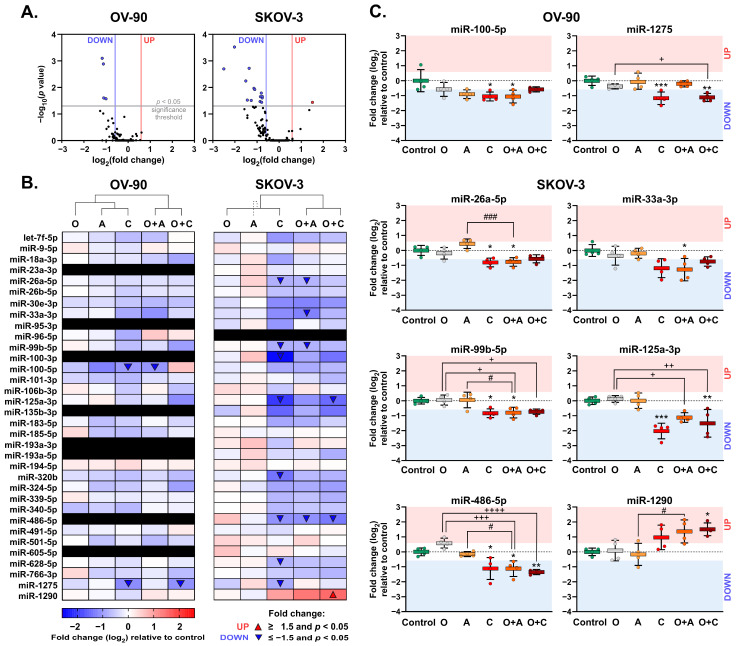
Expression of miRNAs in response to tested inhibitors in SKOV-3 and OV-90 cell lines relative to untreated controls. Cells were treated with O, A, C, or their combinations for 48 h. Levels of miRNAs were determined via real-time qPCR and expressed as means of logarithmic fold change ± SD. (**A**) Volcano plots for miRNAs. Significantly down- and upregulated miRNAs are highlighted with blue and red dots, respectively (absolute fold changes in expression ≥ 1.5 and *p* < 0.05). (**B**) Heatmaps for miRNA expression. Significantly (*p* < 0.05) down- and upregulated miRNAs (absolute fold change ≥ 1.5) are highlighted with blue and red triangles, respectively. Non-informative miRNAs with raw C_T_ values ≥ 33 in ≥75% of control samples (marked as black rectangles) were excluded from the analyses. Hierarchical clustering via heatmaps with the ClustVis web tool was performed using correlation distance and average linkage based on miRNA expression profiles. In SKOV-3 cells, the dashed line shows clustering of O and C groups with an A group. (**C**) Statistical analysis for the expression of significantly dysregulated miRNA assessed with ordinary one-way ANOVA followed by Šídák multiple comparison tests (normally distributed data with homogenous variance) or Kruskal–Wallis followed by Dunn’s multiple comparison test (non-normally distributed data): * *p* < 0.05, ** *p* < 0.01, *** *p* < 0.001 (treatment vs. control); + *p* < 0.05, ++ *p* < 0.01, +++ *p* < 0.001, ++++ *p* < 0.0001 (O vs. combination with A or C); # *p* < 0.05, ### *p* < 0.001 (A or C vs. respective combinations with O). The red and blue areas indicate fold-change values for up- and downregulated miRNAs (absolute log_2_ of fold change ≥ 0.585), respectively.

Of the 45 human miRNAs analyzed, a total of 26 and 33 miRNAs were considered informative in OV-90 and SKOV-3 cells, respectively (Figure 3A,B). Olaparib alone exerted no significant effect on the expression of detected miRNAs in either cell line. Cluster analysis between treatment groups showed notable similarities in the miRNAs between olaparib combination groups compared to single-agent inhibitors (Figure 3B).

In OV-90 cells, two miRNAs were differently expressed (FC ≥ 1.5, *p* < 0.05), all of which were downregulated in response to CHK1i or olaparib combinations (Figure 3A). Olaparib combined with ATRi or CHK1i significantly decreased the expression of miR-100-5p by 2.1-fold and miR-1275 by 2.2-fold compared to untreated cells, respectively (Figure 3B).

In SKOV-3 cells, tested inhibitors or their combinations significantly changed the expression of 10 miRNAs: 9 (miR-26a-5p, miR-33a-3p, miR-99b-5p, miR-100-3p, miR-125a-3p, miR-320b, miR-486-5p, miR-628-5p, and miR-1275) were downregulated by 1.7–2.9-fold, and 1 (miR-1290) was upregulated by 2.9-fold (Figure 3A,B). In total, six (miR-26a-5p, miR-33a-3p, miR-99b-5p, miR-125a-3p, miR-486-5p, and miR-1290) unique miRNAs were differentially expressed in response to olaparib combined with ATR/CHK1 pathway inhibition (Figure 3C). The addition of ATRi to olaparib significantly decreased the expression of three miRNAs (miR-26a-5p, miR-33a-3p, and miR-99b-5p) compared to the control cells. Combining olaparib with CHK1i resulted in the downregulation of two miRNAs (miR-125a-3p and miR-486-5p) and upregulation of miR-1290 (Figure 3B). The expression levels of significantly dysregulated miRNAs in response to olaparib combinations are shown in Figure 3C. The results of miRNA expression analysis for all informative miRNAs are presented in Supplementary Figures (Figures S2 and S3).

On the whole, targeted inhibition of the ATR/CHK1 pathway combined with olaparib induced differential expression of a few miRNAs in OV-90 and SKOC-3 cell lines, highlighting potential changes in the post-translational regulation of gene expression associated with the cytotoxic activity of tested combinations in OC cells.

### 2.4. Regulatory miRNA-mRNA Networks and Functional Enrichment Analysis

miRNAs showing significant dysregulation in response to olaparib combined with ATRi or CHK1 in OV-90 and SKOV-3 cells were selected for further bioinformatic analysis.

**Figure 4:**
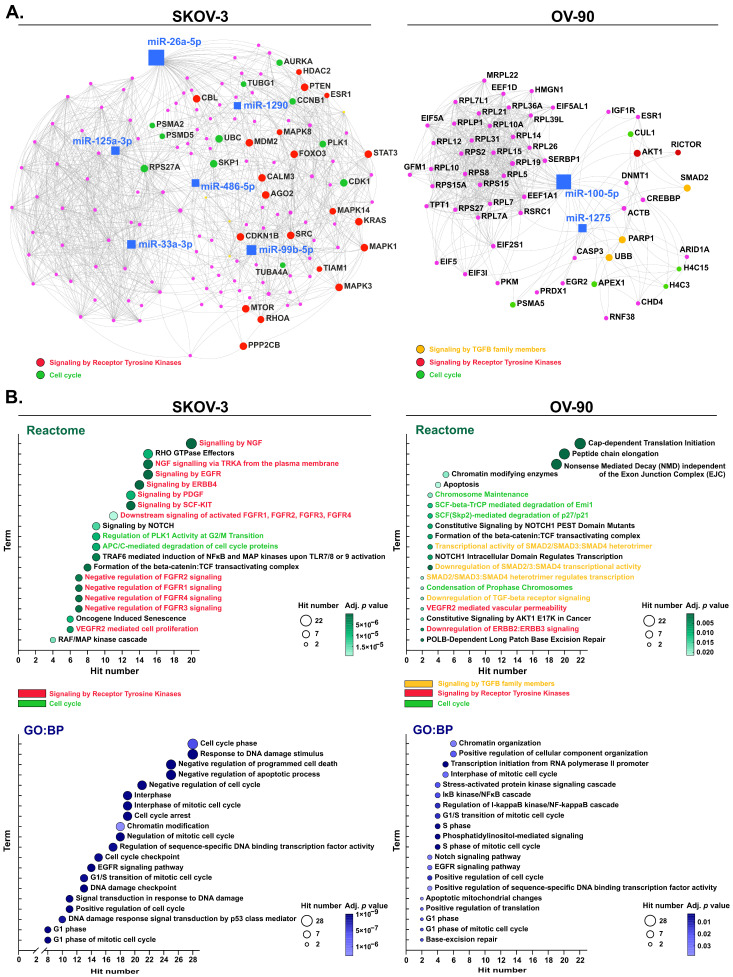
Network-based analyses for target genes of dysregulated miRNAs in response to olaparib combinations with ATRi or CHK1i in SKOV-3 and OV-90 cell lines. (**A**) The miRNA–mRNA regulatory network with PPIs highlighting genes associated with selected enriched Reactome terms in different colors as indicated. The remaining target genes are represented by pink circular nodes. (**B**) A bubble blot showing selected Reactome and GO:BP terms from the functional enrichment analysis. The terms were ranked based on the target gene hit number and significance level (adjusted *p*-value).

First, miRNA-mRNA regulatory networks with protein–protein interactions (PPIs) interactions were created for miRNAs differentially expressed in OC cell lines and their experimentally confirmed target genes (Figure 4A). In OV-90 cells, two dysregulated miRNAs were predicted to regulate 103 genes, including *PARP1* targeted by miR-100-5p and miR-1275. Six miRNAs differently expressed in SKOV-3 cells were shown to target 1380 genes; however, none of them targeted the gene encoding PARP1 (Figure 4A).

Next, associations of target genes for dysregulated miRNAs with specific pathways were revealed by functional enrichment analyses. Genes linked to signal transduction pathways and cell cycle were highlighted in regulatory networks since most of the enriched terms were associated with these processes (Figure 4A).

The most significantly enriched Reactome terms in OV-90 cells included “Chromatin-modifying enzymes”, “Apoptosis”, and a few pathways associated with translation, signaling by receptor tyrosine kinases (RTKs) as well as signaling by TGFβ family members and cell cycles (Figure 4B). Targeting of the *PARP1* gene was associated with “downregulation of SMAD2/3:SMAD4 transcriptional activity” and “POLB-Dependent Long Patch Base Excision Repair”.

In SKOV-3 cells, genes targeted by differently expressed miRNAs were also predicted to be mostly involved in a few pathways associated with signaling by RTKs and cell cycles based on the results provided by Reactome (Figure 4B). Pathways involved in signaling by Rho GTPases, NOTCH, Wnt, and RAF/MAP kinase cascade were also significantly enriched (Figure 4B). The results obtained with the GO database were like enriched pathways detected with Reactome in the context of the regulation of cell cycles and programmed cell death (Figure 4B).

Altogether, functional enrichment analyses revealed pathways and biological processes likely associated with differentially expressed miRNAs in OC cell lines experiencing increased cell death in response to olaparib through ATR/CHK1 pathway inhibition.

Moreover, the mRNA levels of *CASP3* and *H2AX* and protein expression of cytochrome c after treatment with the tested inhibitors at 4 µM concentrations were examined (Figure S1A,B). The disruption of mitochondrial membrane integrity, and indirectly the release of cytochrome c, is crucial for apoptosis. After 24 h of incubation, no significant changes in cytochrome c expression levels were observed in the SKOV-3 cell line. After 48 h of incubation, both combinations increased protein expression by approximately 2.5-fold in the SKOV-3 line, compared to an approximately 1.5-fold increase after PARPi and CHK1i and an approximately 2-fold increase after incubation with ATRi. As in the OV-90 line, after 24 h of incubation, an approximately 1.5-fold increase in the expression of the protein was observed after treatment with the PARPi + ATRi combination. In contrast, the changes observed after 48 h of incubation in this cell line were negligible. The mRNA levels of *H2AX* were not significantly changed, whereas *CASP3* mRNA levels were elevated around 1.5-fold only after olaparib treatment in the OV-90 cell line.

## 3. Discussion

EMT is considered a key factor in cancer progression, particularly in epithelial-derived tumors, such as OC. An immunohistological analysis of primary and metastatic ovarian carcinomas indicated a strong association between EMT, peritoneal metastasis, and patient survival [19]. Additionally, gene expression studies showed that metastatic ovarian tumors often display mesenchymal characteristics, further linking EMT to the aggressiveness and poor prognosis of EOC [20]. However, the mechanisms by which the combination of olaparib and ATRi/CHK1i affect EMT in OC are not fully understood.

Five days of treatment with increasing concentrations of olaparib demonstrated dose-dependent cytotoxicity, showing greater efficacy in OV-90 cells than in SKOV-3 cells. Combination treatments revealed promising results, as olaparib with either ATRi or CHK1i acted synergistically, significantly reducing cell viability, especially in OV-90 cells, as formerly proven [21,22].

Additionally, combination therapies effectively inhibited cell migration and invasion, two critical processes for cancer metastasis, particularly with olaparib and CHK1i cotreatment. Reduced levels of matrix metalloproteinases (MMP2 and MMP9), key enzymes in metastatic progression, further supported these findings, as their high levels are correlated with poor survival [16]. Olaparib alone reduced mRNA expression levels of *MMP2* and *MMP9* in oral carcinoma cell lines [23] and, combined with bevacizumab, was found to reduce the serum levels of MMP9 in advanced colorectal cancer [24]. Furthermore, transcription factors like Snail regulate chemoresistance, stemness acquisition, and spheroid formation in OC cells and their upregulation, followed by the downregulation of E-cadherin, enhance the invasiveness of OC [25]. Snail expression levels had a decreasing tendency after combination treatment in both cell lines. Treatment with the PARP inhibitors PJ-34 and KU0058948 downregulated endogenous Snail in human melanoma cell lines A375 and G361 [26]. In addition, high N-cadherin protein expression in OC triggers cell invasion and is also correlated with poor survival [27]. N-cadherin levels were shown to have a downward tendency after treatment in the SKOV-3 cell line and decreased significantly, followed by the upregulation of E-cadherin levels in OV-90 cells. The downregulation of N-cadherin following olaparib treatment in other cancer types, such as lung, cervical, and bone cancer, supports the notion that olaparib can influence the EMT process [28].

The activation of the EMT program in cancer cells results in increased invasive and metastatic properties as well as multidrug resistance (MDR). In our study, we have shown that, despite a slight increase in *ABCB1* mRNA levels, it did not affect the MDR1 protein expression levels in both cell lines.

The disruption of apoptosis can lead to uncontrolled cell growth and the development of associated diseases [29]. Thus, as we have proven inhibitor combinations’ cytotoxicity and have previously shown that they induce apoptosis, we decided to examine cytochrome c expression. Olaparib treatment at a concentration of 4 µM was not sufficient to significantly increase cytochrome c expression. However, after 48 h, both combinations increased cytochrome c release statistically significantly only in the SKOV-3 line.

In OC, certain miRNAs are associated with malignancy, playing roles in tumor progression and the epithelial–mesenchymal transition. miRNA analysis revealed that olaparib combined with ATRi or CHK1i altered the expression of multiple miRNAs linked to cell cycle and apoptosis pathways, potentially enhancing cytotoxic effects and limiting the migratory and invasive abilities of OC cells.

For instance, miR-100-5p expression was downregulated after CHK1i or O+A treatment in the OV-90 cell line. In a related study, long non-coding RNA (lncRNA) SDCBP2-AS1 and ependymin-related protein 1 (EPDR1) levels were suppressed, while miR-100-5p was elevated in OC. Following the upregulation of lncRNA SDCBP2-AS1 or EPDR1 OC cell viability, migration and invasion were diminished, and the apoptosis rate was increased. Then, it was illustrated that lncRNA SDCBP2-AS1 regulates EPDR1 through the suppression of miR-100-5p to inhibit OC development and progression [30]. Conversely, miR-1275, which was downregulated after C and O+C treatment in OV-90 cells, was found to have a tumor-suppressive role in breast cancer [31].

miR-486-5p expression was significantly reduced after O+C treatment in SKOV-3 cells. This miRNA was found to be over-expressed in serum and ascite samples from endometriosis-associated OC patients, and its downregulation in immortalized ovarian endometrioma cells was shown to inhibit cell proliferation and migration [32]. Additionally, miR-486-5p targets OLFM4, a gene whose downregulation in ovarian serous adenocarcinoma tissues contributes to enhanced proliferation, migration, and invasion of OC cells. The upregulation of OLFM4 expression, regulated by miR-486-5p, slowed the progression and development of OC [33]. MiR-125a-3p, which was downregulated after C and O+C treatment, has been linked to olaparib sensitivity, and its over-expression inhibited the invasion and migration of A2780 and OVCAR-3 cell lines [34]. miR-26a-5p, which was also downregulated following treatment with C and O+A inhibitors, has been found to be upregulated in bladder cancer patients [35]. Similarly, miR-99b-5p was upregulated in colorectal cancer [36], while miR-1290 was associated with various types of cancer, including colorectal, pancreatic, and cervical cancers [37]. In cancer-associated fibroblasts (CAFs), miR-1290 over-expression was correlated with changes in EMT markers and the promotion of mTOR and Akt phosphorylation within ovarian carcinoma cells, which ultimately increased tumor growth [38]. On the other hand, another study confirmed that miR-1290 acted as a tumor suppressor gene in gynecologic tumors and its suppression could promote the proliferation of OC cells [39]. Although we found miR-1290 upregulation after O+C treatment in the SKOV-3 cell line, it was not associated with changes in cell viability, migration, or invasion. Moreover, expression levels of MMP2 and MMP9, known to play key roles in tumor metastasis, decreased following O+C treatment.

Bioinformatics analysis of these miRNAs indicated associations with chromatin modification, RTK signaling, and TGFβ-related pathways, suggesting mechanisms by which combined treatment could disrupt cell survival processes. Altogether, this study underscores the promise of combining PARP inhibitors with ATR/CHK1 pathway inhibition to reduce OC cell viability and invasiveness, potentially overcoming the limitations of single-agent therapies in the treatment of epithelial ovarian cancer.

## 4. Materials and Methods

### 4.1. Reagents

Culture media (RPMI 1640, DMEM), heat-inactivated fetal bovine serum (FBS), and trypsin-EDTA were obtained from Gibco (Thermo Fisher Scientific, Waltham, MA, USA). PARPi (AZD2281, olaparib, O) was purchased from MedChemExpress (Monmouth Junction, NJ, USA). ATRi (AZD6738, ceralasertib, A) and CHK1i (MK-8776, C) were purchased from Wuhan ChemNorm Biotech (Wuhan, China). The stock solutions of inhibitors were stored at −80 °C for a maximum of 6 months and were prepared by dissolving them in 100% dimethyl sulfoxide (DMSO). Other chemicals and solvents were of high analytical grade and were obtained from Merck Life Science (Poznań, Poland) or Avantor Performance Materials Poland S.A. (Gliwice, Poland).

### 4.2. Cell Culture and Drug Administration

A SKOV-3 cell line with a loss of *TP53* function (*TP53* null) (human ovarian adenocarcinoma, HTB-77) and a human OV-90 cell line with a mutated *TP53* gene (human malignant papillary serous carcinoma, CRL-11732™) were purchased from American Type Culture Collection (ATCC, Rockville, MD, USA). The cells were cultured in monolayers either in RPMI 1640 medium (SKOV-3) or DMEM (OV-90) containing GlutaMAX™ supplement, HEPES, and 10% FBS in a cell culture incubator with an atmosphere of 5% CO_2_ at 37 °C. During this study, the cells were thawed and passaged within 2 months of each experiment and regularly checked for mycoplasma contamination. The cells were treated with 10 μM PARPi (O), 5 μM ATRi (A), and 2.5 μM CHK1i (C) during all experiments, as chosen based on the CDI values, except quantitative real-time PCR, during which the cells were treated with tested inhibitors at a concentration of 4 μM, in order to compare them with our previous studies of protein expression [22]. Unless stated otherwise, each experiment was independently repeated three times (n = 3).

### 4.3. MTT Assay

After 5 days of treatment with the indicated doses of tested inhibitors, cell viability was evaluated with an MTT assay. Logarithmically growing cells (1 × 10^4^) were seeded into 96-well plates. After treatment, the medium was aspirated, the cells were washed with PBS, and then 50 μL of MTT solution (0.5 mg/mL in DPBS) was added to each well, and the plates were incubated for 4 h (37 °C, 5% CO_2_). Then, 100 µL of DMSO was added per well. After the complete solubilization of violet formazan crystals, formed as a result of MTT reduction within metabolically viable cells, the samples were mixed for about 30 s using a plate shaker, and the absorbance was measured at 580 nm and 720 as a reference wavelength, using a microplate reader (Awareness Technology Inc., Palm City, FL, USA) [40]. Several concentration ratios of the compounds were tested to select the most effective ratio of drug combinations (O+A or O+C). To analyze the drug interactions between olaparib combined with either ATRi or CHK1i, CDI was calculated as described previously, according to the formula CDI = AB/(A × B) [41]. In compliance with the absorbance of each group, AB is the cell viability ratio of the two-drug combination group to the untreated control group, and A or B is the ratio of the single-drug group to the control group. CDI values below 1.00 indicate a synergistic effect, CDI < 0.7 significant synergism, CDI = 1 additive effect, and CDI > 1.00 antagonistic.

### 4.4. Cell Migration Assay

Cell migration was measured by a wound healing assay. Cells were seeded into a 6-well plate at a density of 7 × 10^4^ cells/well. After overnight incubation, the confluent cell monolayers were wounded via scratching with a 200 μL pipette tip and then treated with indicated concentrations of inhibitors. Cell migration was tracked using an inverted phase-contrast microscope Olympus IX70 microscope (Olympus, Tokyo, Japan) using a 10× objective, and images of the scratches were captured from 0 to 48 h in SKOV-3 cells and from 0 to 96 h in OV-90 cells. The size of the wound was analyzed using CellD software v1.16 (Olympus, Tokyo, Japan).

### 4.5. Invasion Assay

For the Boyden chamber invasion assay, 1  ×  10^4^ cells were seeded into the Thincert cell culture insert (Greiner, cat. no. 662638) coated with a layer of extracellular matrix (ECM) gel (Geltrex™, Gibco™, Thermo Fisher Scientific, Waltham, MA, USA) and incubated overnight. Following cell attachment, inserts were removed, and the cells were washed twice with PBS. The culture medium (with 10% FBS) was added to the lower chamber, and the cells were cultured in a 2% FBS-containing medium following treatment with the inhibitors and allowed to migrate for a designated time (48 h), followed by cell fixation in 4% paraformaldehyde for 30 min. Then, the cells were permeabilized by 90% methanol, stained with 0.5% (*w*/*v*) crystal violet, and washed with PBS to remove excess stain. Non-invasive cells on the top surface of the membrane were scraped off using cotton swabs. Invasive cells on the lower surface were examined with an inverted phase-contrast microscope Olympus IX70 microscope using a 10× objective. The dye was then extracted using 30% acetic acid, and the absorbance was determined spectrophotometrically on a microplate reader (Synergy HTX, BioTek, Shoreline, WA, USA) at an experimental wavelength of 590 nm. Cell invasiveness was calculated as the percentage of untreated control cells using absorbance values.

### 4.6. Western Blot Analysis

The cells were seeded in 100 mm dishes (2.0 × 10^6^ cells) to analyze protein expression levels and then treated for 72 h. For MDR1 and cytochrome c expression level investigation, the cells were treated with the tested inhibitors at a concentration of 4 μM for 24 and 48 h, in order to compare them with mRNA levels measured with qPCR. Following the treatment, the whole-cell lysates were prepared in ice-cold RIPA buffer (Thermo Fisher Scientific, Waltham, MA, USA) supplemented with phenylmethylsulfonyl fluoride (1 mM PMSF, Merck Life Science (Poznań, Poland)), Halt Protease Inhibitor Cocktail (Thermo Fisher Scientific), and Halt Phosphatase Inhibitor Cocktail (Thermo Fisher Scientific). Then, the samples were sonicated and centrifuged, and mPAGE 4X LDS Sample Buffer supplemented with 50 mM β-mercaptoethanol (Merck Life Science (Poznań, Poland)) was used as a sample buffer. A total of 30 μg of proteins was loaded into each lane of the mPAGE Bis-Tris gels, and then, the proteins were separated by SDS polyacrylamide gel electrophoresis at 180 V for 30 min in an electrophoresis tank (Mini-PROTEAN Tetra Cell, Bio-Rad, Hercules, CA, USA) using MOPS SDS running buffer, Merck Life Science (Poznań, Poland). Next, the proteins were transferred onto 0.45 µm PVDF membranes using a semi-dry transfer Trans-Blot Turbo Transfer System (Bio-Rad, Hercules, CA, USA) in mPAGE Transfer Buffer according to the mPAGE Bis-Tris gel manufacturer’s optimized instructions. After blocking nonspecific sites with 5% non-fat dry milk in TBST for 1 h and washing with TBST, the membranes were incubated overnight at 4 °C with rabbit monoclonal antibodies (1:1000) against MMP9 (cat. no.: #13667), MMP2 (cat. no.: #40994), E-cadherin (cat. no.: #3195), N-cadherin (cat. no.: #13116), Snail (cat. no.: #3879) and Cytochrome C (cat. no. #11940) from Cell Signaling Technology, Inc. (Danvers, MA, USA), MDR1 (Invitrogen™ (Thermo Fisher Scientific), cat. no. MA5-32180), and mouse monoclonal anti-β-actin antibody (cat. no. A1878, Merck Life Science (Poznań, Poland)), followed by incubation with anti-rabbit IgG horseradish peroxidase-conjugated (cat. no.: 7074, Cell Signaling Technology) or anti-mouse IgG HRP-conjugated (cat. no.: A28177, Invitrogen, Thermo Fisher Scientific, Waltham, MA, USA) secondary antibodies for 1 h at room temperature. To obtain a chemiluminescence signal, enhanced chemiluminescent (ECL) substrates were used: a SuperSignal™ West Pico PLUS Chemiluminescent Substrate or SuperSignal™ West Atto Ultimate Sensitivity Substrate, for low-abundance proteins (Thermo Fisher Scientific, Waltham, MA, USA). Images were visualized using a c300 Azure imaging system (Azure Biosystems, Dublin, CA, USA). Densitometric quantification of the immunoreactive bands was performed with ImageJ software v1.5. Relative protein levels were expressed as the ratio of the densitometric volume of the tested band to that of the respective β-actin band.

### 4.7. RNA Isolation, cDNA Synthesis, and Quantitative Real-Time PCR

*ACTB* (Assay ID: Hs01060665_g1), *ABCB1* (Assay ID: Hs00184500_m1)*, ATR* (Assay ID: Hs00992123_m1)*, CASP3* (Assay ID: Hs00234387_m1)*, CHEK1* (Assay ID: Hs00967506_m1)*, H2AX* (Assay ID: Hs00266783_s1), and *PARP1* (Assay ID: Hs00242302_m1) gene expression profiling was conducted using quantitative real-time PCR (qRT-PCR). Cells were seeded in 100 mm dishes (2 × 10^6^ cells) and treated with tested inhibitors at a concentration of 4 μM for 48 h. Afterwards, total RNA was isolated and purified with a mirVana™ miRNA Isolation Kit with phenol (Invitrogen™, Thermo Fisher Scientific). Absorbance measurements at 230, 260, and 280 nm using a BioTek Eon™ microplate spectrophotometer were analyzed to ensure the quality and quantity of isolated RNA (BioTek, Winooski, VT, USA). A HighCapacity cDNA Reverse Transcription Kit (Applied Biosystems™, Thermo Fisher Scientific) with RNase inhibitor was used for reverse transcription for a total of 1000 ng of RNA, and the reaction took place using a PTC-200 DNA Engine^®^ Cycler (MJ Research Inc., Saint-Bruno-de-Montarville, QC, Canada). Afterwards, 10 ng of the obtained cDNA was used for a qRT-PCR reaction. TaqMan™ Universal Master Mix II with no UNG and predesigned TaqMan™ Gene Expression Assays (Applied Biosystems™, Thermo Fisher Scientific) were used; the reaction took place in a final volume of 10 μL and was run in the Rotor-Gene Q 5plex HRM (QIAGEN Inc., Hilden, Germany). Q-Rex software, v2.0 (QIAGEN Inc., Germany) was used to analyze the raw data and, using ACTB as a reference gene, to calculate relative mRNA expression by the comparative 2^−∆∆Ct^ method [42]. The results are presented as a fold change (FC) relative to untreated control cells. n = 4, with each sample run in duplicate or triplicate (SD between technical replicates ≤ 0.3 cycles). The results are presented as mean ± SD.

### 4.8. RNA Isolation and RT-qPCR Using Custom TaqMan™ Array MicroRNA Cards

RT-qPCR using Custom TaqMan™ Array MicroRNA Cards (Thermo Fisher Scientific, Waltham, MA, USA) was performed as described previously [43]. Initially, global profiling of 754 human miRNAs using TaqMan™ Array Human MicroRNA A+B Cards v3.0 (Thermo Fisher Scientific, Waltham, MA, USA) in PEO1 and PEO1-OR cell lines was performed. Dysregulated miRNAs were prioritized through bioinformatics analyses using the MIENTURNET web tool [44] and miRTarBase database [45], based on strong experimental evidence of miRNA–target interactions and network node degree. From this, 45 relevant miRNAs were selected and validated using Custom TaqMan™ Array MicroRNA Cards with two endogenous controls (U6 snRNA and RNU48 snoRNA). Briefly, after 48 h of treatment, a mirVana™ miRNA Isolation Kit (Thermo Fisher Scientific, Waltham, MA, USA) was used. The reverse transcription took place with 1000 ng of total RNA and was run in a PTC-200 DNA Engine^®^ Cycler (MJ Research Inc., St. Bruno, QC, Canada), using a TaqMan™ MicroRNA Reverse Transcription Kit and Custom RT Primer Pool composed of individual RT primers for each target provided with the Custom TaqMan™ Array MicroRNA Cards. Total RNA from 4 independent experiments was then used (n = 4). Then, 200 ng of cDNA was used together with 2× TaqMan™ Universal Master Mix II with no UNG, and the qPCR reactions were run in a 7900HT Fast Real-Time PCR System (Thermo Fisher Scientific, Waltham, MA, USA). DataAssist™ v3.01 software was used for exporting raw C_T_ values collected using automatic baseline settings and a threshold of 0.2 C_T_ values ≥ 33 for miRNAs in treated samples were included in the calculations. For data processing, miRNAs were considered informative (detected) when C_T_ values were below 33 for at least three out of four biological replicates in untreated control samples. Two endogenous control assays (U6 snRNA in duplicate, RNU48 snoRNA) were used for the calculations of relative miRNA expression, which was then calculated as FC compared to untreated cells using the comparative 2^−∆∆Ct^ method, and log-transformed relative quantity data (log2 of FC) were used for statistical analysis. A heatmap created with GraphPad Prism v9.5.1 (GraphPad Software, San Diego, CA, USA) was deployed to visualize the expression of all informative miRNAs. Significantly dysregulated miRNAs were defined as differentially expressed (up- or downregulated) with an absolute FC ≥ 1.5 (log_2_ of FC ≥ 0.585) and *p* < 0.05.

### 4.9. miRNA–mRNA Regulatory Networks

The mapping of dysregulated miRNAs to target genes, and the generation of the miRNA–mRNA network was performed using miRNet 2.0, a web-based platform [46] (accessed on 21 August 2024), employing two up-to-date miRNA target gene databases with experimentally validated interactions (miRTarBase v9.0 and TarBase v9.0). The network was customized by PPIs based on the STRING interactome (confidence score cut-off of 800 in experimentally derived interactions) and reducing the network density using the shortest path filter. The resulting network was visualized in miRNet 2.0.

### 4.10. Functional Annotation and Pathway Enrichment Analysis

Functional enrichment analysis was performed in miRNet 2.0 at the target gene level by a hypergeometric test algorithm using the Gene Ontology Biological Process (GO:BP) and Reactome pathway databases. A threshold of an adjusted *p*-value < 0.05 was used to define statistically significant terms.

### 4.11. Statistical Analysis

Data are presented as the mean ± SD of at least three independent experiments. Statistical analyses were performed with two-way and one-way ANOVA, with the Tukey post hoc test for multiple comparisons, as appropriate (StatSoft, Tulsa, OK, USA). *p*-values < 0.05 were considered statistically significant.

## 5. Conclusions

EMT is a pivotal mechanism in the progression and metastasis of OC, driven by transcription factors such as Snail and influenced by key molecular pathways. This study highlights the potential of combining PARP inhibitors like olaparib with ATR or CHK1 inhibitors to enhance therapeutic efficacy in *TP53*-mutated OC. These combination treatments not only demonstrated synergistic cytotoxic effects but also inhibited critical processes like cell migration and invasion, reduced expression of metastasis-associated proteins (MMP2, MMP9, and cadherin switch), and modulated EMT-related transcription factors. Furthermore, miRNA analysis revealed significant alterations in expression profiles linked to tumor progression and apoptosis, providing insights into the molecular mechanisms behind these therapies. Overall, this research supports targeting multiple pathways to overcome the limitations of monotherapies and improve outcomes in epithelial ovarian cancer.

## Data Availability

The data presented in this study are available on request from the corresponding author.

## Supplementary Materials

The following supporting information can be downloaded at https://www.mdpi.com/article/10.3390/ijms26020693/s1.
