# Supplementary material for: Olaparib Combined with DDR Inhibitors Effectively Prevents EMT and Affects miRNA Regulation in TP53-Mutated Epithelial Ovarian Cancer Cell Lines
Source: Int J Mol Sci. 2025 Jan 15;26(2):693. doi: 10.3390/ijms26020693 (PMC11766100; doi:10.3390/ijms26020693)
Supplement: Supplementary file 1 [file ijms-26-00693-s001.zip › ijms-3374068-supplementary.pdf]

Olaparib Combined with DDR Inhibitors Effectively Prevents EMT and Affects miRNA Regulation in *TP53*-Mutated Epithelial Ovarian Cancer Cell Lines

Patrycja Gralewska [1], Łukasz Biegała [1], Arkadiusz Gajek [1], Izabela Szymczak-Pajor [2], Agnieszka Marczak [1], Agnieszka Śliwińska [2] and Aneta Rogalska [1,*]

[1] **Department of Medical Biophysics, Institute of Biophysics, Faculty of Biology and Environmental Protection, University of Lodz, 141/143 Pomorska Street, 90-236 Lodz, Poland; patrycja.gralewska@biol.uni.lodz.pl (P.G.); arkadiusz.gajek@biol.uni.lodz.pl (A.G.); agnieszka.marczak@biol.uni.lodz.pl (A.M.)**

[2] **Department of Nucleic Acid Biochemistry, Medical University of Lodz, 251 Pomorska Street, 92-213 Lodz, Poland; izabela.szymczak@umed.lodz.pl (I.S.P.); agnieszka.sliwinska@umed.lodz.pl (A.Ś.)**

[*] **Correspondence: aneta.rogalska@biol.uni.lodz.pl; Tel.: +48-42-635-44-81**

The experiments were performed as described in sections 4.6, 4.7, and 4.8 of the main article.

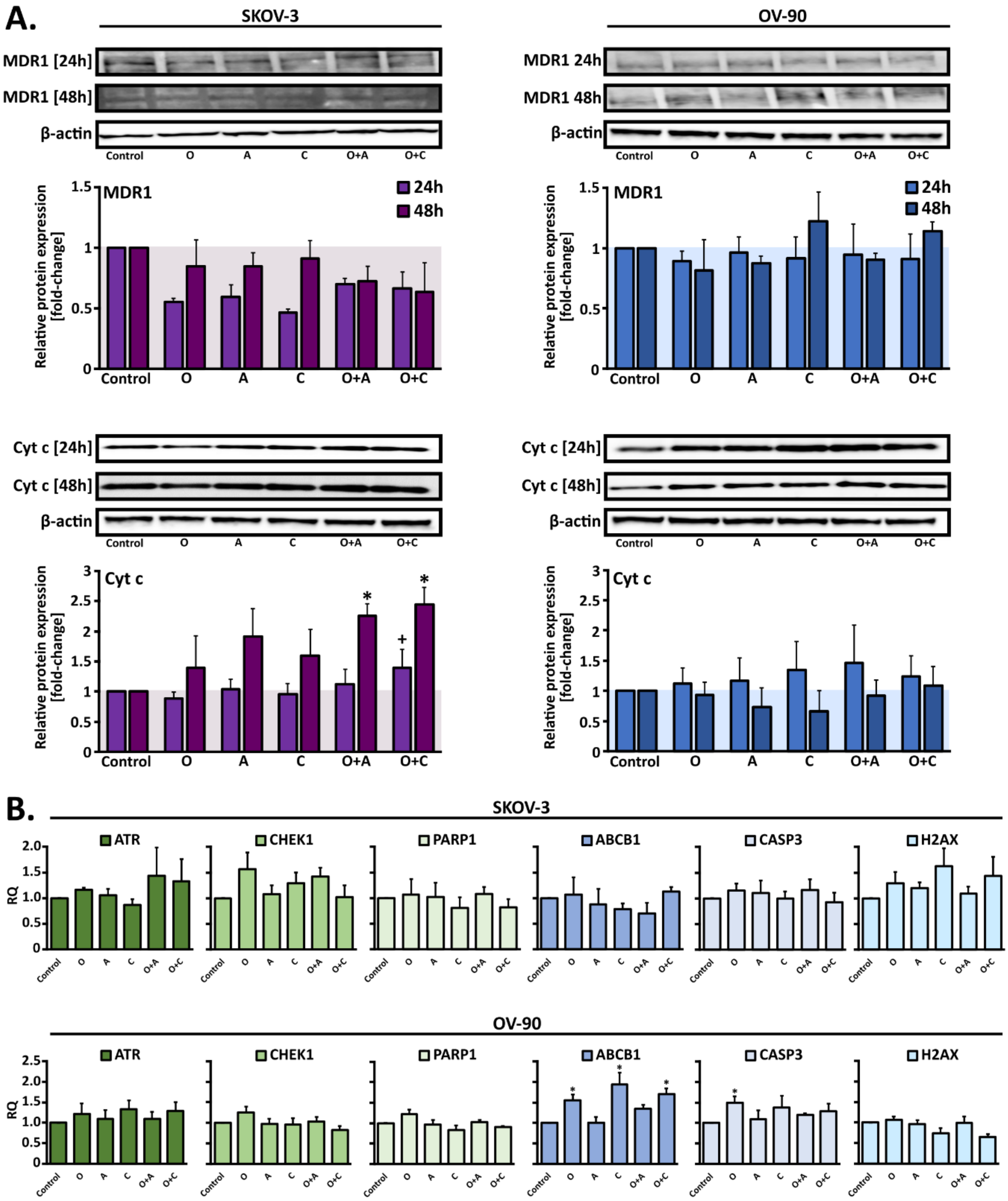

Supplementary Figure S1. Olaparib combined with ATRi or CHK1i at 4 µM does not significantly affect the MDR1 and cytochrome c protein expression levels and mRNA levels of the corresponding genes in SKOV-3 and OV-90 cells (A) Relative expression and representative western blot images of MDR1 and cyt c in SKOV-3 and OV-90 cells (n=3). * treatment vs. control ($p < 0.05$). + olaparib vs. combination treatment (O+A; O+C) ($p < 0.05$). **(B)** qRT-PCR analysis of *ABCB1, ATR, CASP3, CHEK1*, *H2AX* and *PARP1* mRNA. Relative mRNA expression was calculated using the $2^{-\Delta\Delta Ct}$ method and β-actin (*ACTB*) as a reference gene (n = 4), * ($p < 0.05$, treatment vs. control)

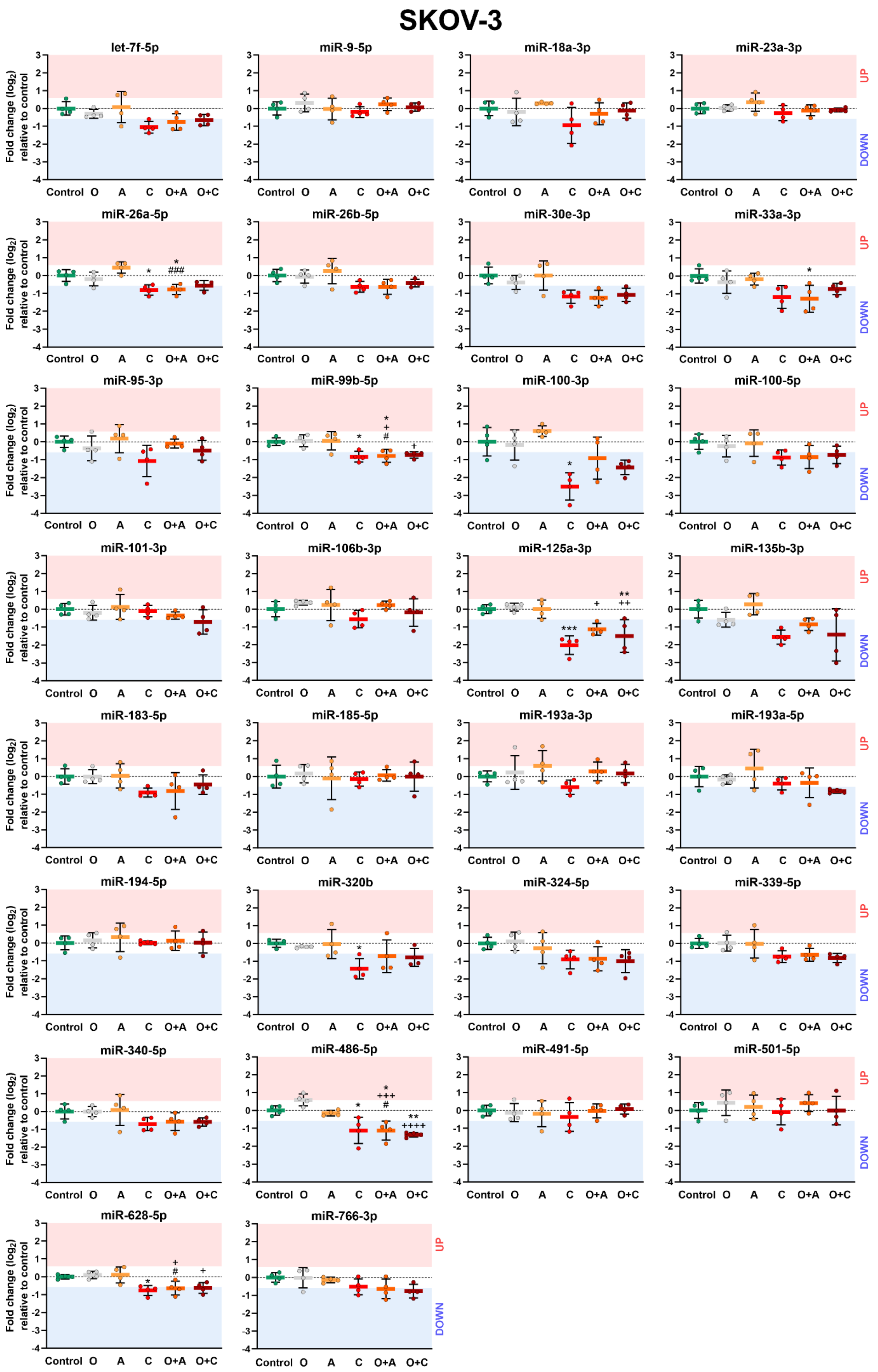
SKOV-3
let-7f-5p
miR-9-5p
miR-18a-3p
miR-23a-3p
miR-26a-5p
miR-26b-5p
miR-30e-3p
miR-33a-3p
miR-95-3p
miR-99b-5p
miR-100-3p
miR-100-5p
miR-101-3p
miR-106b-3p
miR-125a-3p
miR-135b-3p
miR-183-5p
miR-185-5p
miR-193a-3p
miR-193a-5p
miR-194-5p
miR-320b
miR-324-5p
miR-339-5p
miR-340-5p
miR-486-5p
miR-491-5p
miR-501-5p
miR-628-5p
miR-766-3p
Fold change (log2) relative to control
Control O A C O+A O+C
UP
DOWN

Supplementary Figure S2. Overview of the expression of informative miRNA in SKOV-3 cell line. (CT values < 33 in ≥ 75% of control samples) presented as means of logarithmic fold change ± SD (n = 4) in SKOV-3 cell line. Statistical significance was assessed with ordinary one-way ANOVA followed by Šídák multiple comparison tests (normally distributed data with homogenous variance) or Kruskal-Wallis followed by Dunn's multiple comparison test (non-normally distributed data): * $p < 0.05$, ** $p < 0.01$, *** $p < 0.001$ (treatment vs. control); + $p < 0.05$, ++ $p < 0.01$, +++ $p < 0.001$, ++++ $p < 0.0001$ (O vs. combination with A or C); # $p < 0.05$, ### $p < 0.001$ (A or C vs. respective combinations with O). The red and blue areas indicate fold-change values for up- and downregulated miRNAs (absolute log2 of fold change ≥ 0.585), respectively.

OV-90

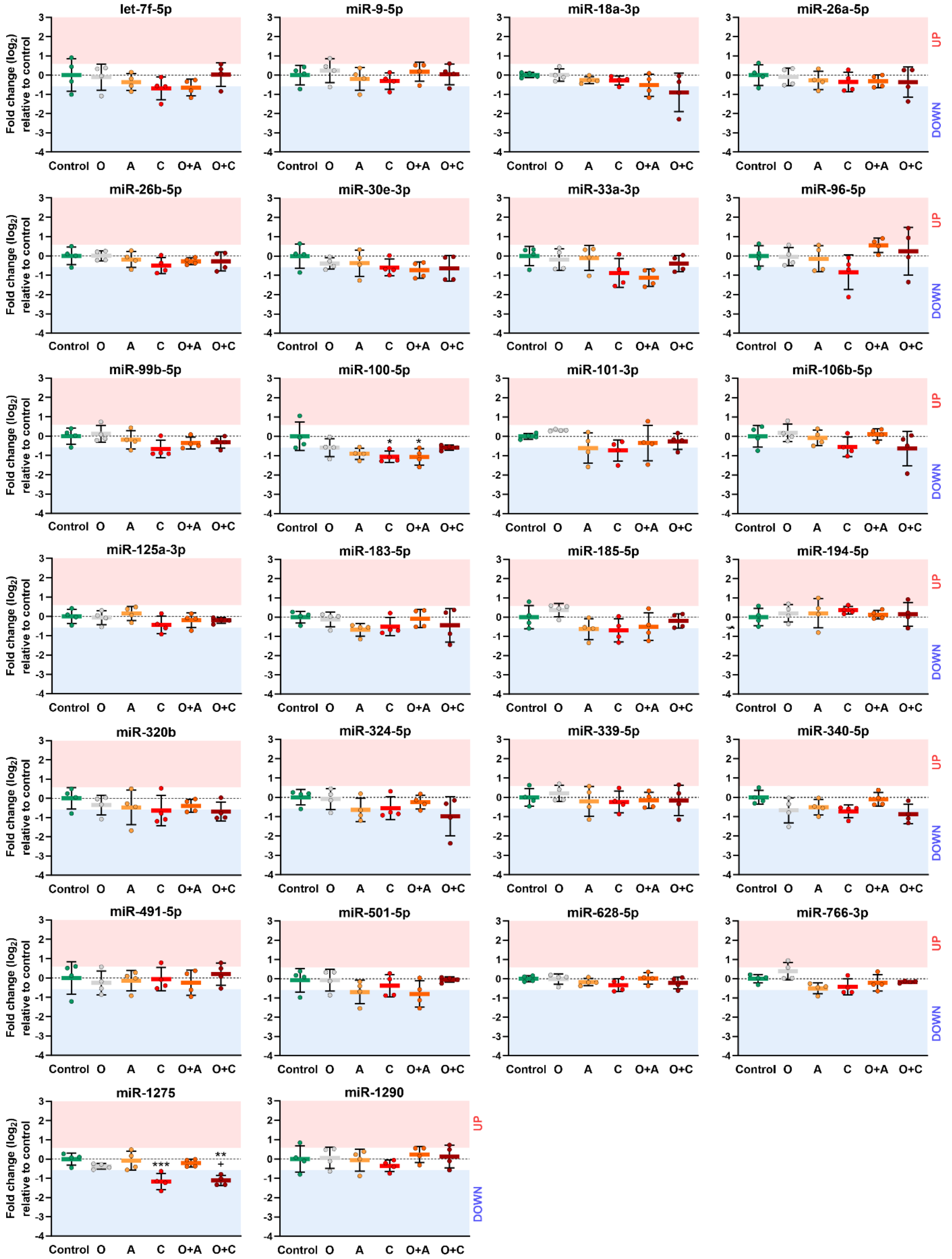

Supplementary Figure S3. Overview of the expression of informative miRNA in OV-90 cell line. (CT values < 33 in ≥ 75% of control samples) presented as means of logarithmic fold change ± SD (n = 4) in OV-90 cell line. Statistical significance was assessed with ordinary one-way ANOVA followed by Šídák multiple comparison tests (normally distributed data with homogenous variance) or Kruskal-Wallis

followed by Dunn's multiple comparison test (non-normally distributed data): * $p < 0.05$, ** $p < 0.01$, *** $p < 0.001$ (treatment vs. control). The red and blue areas indicate fold-change values for up- and downregulated miRNAs (absolute log2 of fold change ≥ 0.585), respectively.
